# Repression of JAK2-STAT1 and PD-L1 by CEP-33779 ameliorates the LPS-induced decline in phagocytic activity of alveolar macrophages and mitigates lung injury in mice

**DOI:** 10.3389/fimmu.2024.1472425

**Published:** 2024-11-26

**Authors:** Yu-Han Wang, A-Guo Li, Hong-Yan Wang, Yong-Sheng Tu

**Affiliations:** ^1^ Guangdong Provincial Key Laboratory of Protein Modification and Degradation, Guangzhou Medical University, Guangzhou, China; ^2^ Department of Physiology, College of Basic Medical Sciences, Guangzhou Medical University, Guangzhou, China; ^3^ The Second Clinical College, Guangzhou Medical University, Guangzhou, China; ^4^ Department of Pathology, College of Basic Medical Sciences, Guangzhou Medical University, Guangzhou, China

**Keywords:** acute lung injury, alveolar macrophage, PD-L1, CEP-33779, phagocytosis

## Abstract

**Background:**

The role of the JAK2-STAT1/PD-L1 pathway in the phagocytic activity of alveolar macrophages (AMs) during LPS-induced acute lung injury in mice remains poorly understood. This study aims to explore whether the JAK2-STAT1/PD-L1 pathway is upregulated on AMs in LPS-induced mice acute lung injury and to further explore the impact of the JAK2-specific inhibitor CEP-33779 on the LPS-induced impairment of AMs phagocytic activity and lung injury.

**Methods:**

ALI was induced in mice via intratracheal administration of LPS, followed by intragastric administration of JAK2 inhibitor CEP-33779 suspension. Immunohistochemistry was conducted to assess PD-L1 expression in lung tissue, as well as p-JAK2, p-STAT1, and PD-L1 expression on AMs in bronchoalveolar lavage fluid (BALF) using immunofluorescence. Levels of TNF-α and IL-6, as well as protein concentration in BALF, were measured using enzyme-linked immunosorbent assay and Bicinchoninic acid assays, respectively. Hematoxylin-eosin staining and lung injury score were employed to evaluate pathological changes in mouse lungs. Total cell count in BALF was determined using a cell counter. Furthermore, western blot and immunofluorescence was conducted to assess the effect of JAK2 and STAT1 inhibitor on JAK2-STAT1 pathway activation and PD-L1 expression, while confocal microscopy with latex beads rabbit IgG FITC complex was used to observe MH-S cells phagocytic ability.

**Results:**

The study revealed that LPS stimulation triggered the activation of the JAK2-STAT1 pathway and an upregulation of PD-L1 on AMs in both LPS-induced acute lung injury mice and MH-S cell lines. Moreover, treatment with the JAK2 and STAT1 inhibitor effectively reduced the activation of JAK2-STAT1 signaling, downregulated PD-L1 expression on AMs in BALF from LPS-induced ALI mice and LPS-stimulated MH-S cells, and significantly improved the LPS-induced reduction in phagocytic activity in MH-S cells. Most notably, CEP-33779 treatment significantly mitigated the pulmonary inflammatory response and lung injury in mice with LPS-induced ALI.

**Conclusions:**

Collectively, these findings imply that the JAK2-STAT1 pathway plays a role in the upregulation of PD-L1, which in turn is associated with the diminished phagocytic activity in LPS-induced AMs as well as lung injury. Furthermore, our study highlights that CEP-33779 treatment can effectively improve the reduced phagocytic activity of AMs and relieve lung injury induced by LPS through suppression of the JAK2-STAT1/PD-L1 pathway.

## 1 Introduction

Acute lung injury (ALI) or its clinical manifestation, acute respiratory distress syndrome (ARDS), is an acute diffuse and inflammatory lung injury that results in increased pulmonary vascular permeability, elevated lung weight, and loss of aerated lung tissue (1). ARDS accounts for 10% of admissions to intensive care units, with over 3 million patients diagnosed annually. The hospital mortality rates for mild, moderate, and severe ARDS are 34.9%, 40.3%, and 46.1% respectively (2, 3). Various pulmonary insults (such as pneumonia or aspiration) or nonpulmonary insults (such as sepsis, pancreatitis, or trauma) can cause ARDS by inducing the development of nonhydrostatic pulmonary edema (3). The pathological changes of ARDS can be categorized into three phases: an early exudative phase, a subacute proliferative phase, and a late fibrotic phase (4). During the exudative phase, resident alveolar macrophages become activated and release proinflammatory cytokines which recruit neutrophils and monocytes/macrophages, while also activating alveolar epithelial cells and effector T cells to sustain inflammation and tissue damage (5). Despite being relatively common in occurrence, ARDS remains a highly lethal or disabling syndrome.

Phagocytosis by alveolar macrophages (AMs) is crucial for controlling bacterial infections. AMs patrol multiple alveolar spaces and rapidly scavenge inhaled bacteria (6). In the steady state, AMs constitute 90-95% of the cellular content within the alveolar compartment, serving as the first line of defense against respiratory pathogens through their high phagocytic capacity (7, 8). However, it has been reported that murine AMs and monocytes in critically ill patients exhibited impaired capacity to capture bacteria for months after recovery from inflammation (9). The phagocytic activity of cultured AMs from BALF is weakened in a murine model of lipopolysaccharide-induced ALI (10). However, it remains largely unknown about the mechanisms mediating reduction of AMs phagocytic activity in ALI.

PD-L1 acts as a negative regulator of immune responses, the engagement of PD-1 by PD-L1 leads to the inhibition of T cell receptor–mediated lymphocyte proliferation and cytokine secretion (11). ALI due to bacterial, viral, and other infections is caused by excessive and uncontrolled inflammatory reactions leading to lung injury. Previous studies have demonstrated elevated expression of PD-L1 on peritoneal macrophages in septic mice (12), and on monocytes in patients with septic shock (13, 14). However, the changes in PD-L1 expression and its regulatory signaling pathways in AMs during LPS-induced ALI are not well understood. Notably, increased levels of PD-L1 on monocytes from sepsis patients were associated with reduced phagocytic function; however, incubation of whole blood with anti-PD-L1 monoclonal antibody was shown to enhance monocyte phagocytosis (15). Therefore, we hypothesized that PD-L1 may play a negative role in phagocytosis. Nevertheless, the precise involvement of PD-L1 in AMs’ phagocytic activity during ALI remains incompletely understood.

Accumulating evidence has revealed the pivotal role of the JAK2-STAT1 pathway in initiating the inflammatory response in the lung. Upon LPS stimulation, JAK2 undergoes immediate tyrosine phosphorylation in RAW264.7 cell line (16). Systemic inhibition of JAK kinases significantly mitigated LPS-induced lung injury (17). In an animal model of ALI induced by LPS, both JAK2 and STAT1 were found to be activated in the lungs (18). Genetic ablation of STAT1 provided protection against LPS-induced lethality (19). However, it remains unclear how exactly the JAK2-STAT1 pathway is altered on AMs in the mouse ALI model. Is there any involvement of JAK2-STAT1 signaling in PD-L1 upregulation on AMs of a mouse ALI model?

In the present study, we observed an up-regulation of JAK2/STAT1/PD-L1 in freshly isolated BALF AMs in a murine model of LPS-induced ALI and in LPS-stimulated MH-S cell lines. This up-regulation was found to be associated with impaired phagocytic activity in MH-S cells. Moreover, treatment with a JAK2 inhibitor restored the phagocytic capacity of MH-S cell lines and ameliorated LPS-induced lung injury in mice.

## 2 Materials and methods

### 2.1 Mice acute lung injury model and CEP-33779 treatment

Six to eight-week-old specific pathogen-free (SPF) grade male BALB/c mice were obtained from Guangdong Medical Experimental Animal Center (license number SCXK [YUE] 2022-0002). They were housed individually in pathogen-free cages at the Experimental Animal Center of Guangzhou Medical University, under standardized conditions with a room temperature of 20-26°C and humidity of 40-70% following a 12-hour light/dark cycle. The mice had access to water and standard rodent chow ad libitum throughout the protocols. The experimental protocol was approved by the Animal Care and Use Committee of Guangzhou Medical University. After one week of acclimatization, the mice were randomly divided into five groups: control group, acute lung injury (ALI) model group, ALI+PEG300 group, ALI+CEP-33779 25mg/kg group, and ALI+CEP-33779 50 mg/kg group. Prior to the experiment, the mice fasted for six hours and then received intraperitoneal injection of pentobarbital sodium (50 mg/kg) for anesthesia. A stock solution of LPS (2 mg/ml) was prepared using PBS. The mouse ALI model was induced by direct intratracheal instillation of LPS (5 mg/kg), as described in other groups’ protocols (20). In the fourth and fifth groups, CEP-33779 pretreatment was administered two hours before LPS instillation followed by oral administration twice daily within 48 hours after LPS instillation at doses of 25 mg/kg and 50 mg/kg respectively. The ALI+PEG300 group received equal volumes of PBS and PEG300 following a similar procedure as CEP-33779 treatment.

### 2.2 Collection of bronchoalveolar lavage fluid

The lungs were lavaged twice with 0.5 ml of chilled PBS to collect BALF, which was then transferred to a 1.5 ml EP tube placed on ice. The recovery rate of BALF exceeded 80%. After centrifugation at 450 ×g for 5 minutes, the supernatant was collected and stored at -80°C until further use. Bradford’s reagent was utilized to quantify the protein content in BALF, while Mouse TNF-α ELISA KIT and Mouse IL-6 ELISA KIT were employed to detect the levels of TNF-α and IL-6 cytokines.

### 2.3 Cells isolation of BALF

The cells in the alveoli were isolated from BALB/c mice lungs by cannulating the trachea with a blunt 20-gauge needle and then lavaging the lungs ten times with 0.8 ml of chilled PBS/EDTA (5 mM). The BALF was collected in a 15 mL tube, immediately placed on ice, filtered through a 200-mesh sieve, and centrifuged at 450 × g for 5 min. The resulting pellets were washed twice with cold PBS. Erythrocyte lysis buffer (1 mL) was added to the cell pellet to remove red blood cells, followed by another centrifugation at 450 × g for 5 min. Finally, the BALF cell pellets were resuspended in 1 ml of phosphate-buffered saline (PBS). The BALF cells were manually counted using a hemocytometer and used for subsequent immunofluorescence staining.

### 2.4 Cell culture and treatment

Mouse alveolar macrophage cell line MH-S was obtained from ATCC. The cells were cultured in RPMI-1640 medium supplemented with 10% fetal bovine serum, 1% penicillin and streptomycin, and 0.05 mM β-mercaptoethanol in a humidified CO_2_ incubator at 37°C with 5% CO_2_.

### 2.5 Hematoxylin-eosin staining of lung tissue and lung injury score

For histopathological assessment, mouse lungs were dissected and fixed in paraformaldehyde for 24 hours. The lung tissues were then sectioned, embedded in paraffin, and cut into 4 μm sections. Subsequently, the sections underwent routine dewaxing using xylene I and xylene II solutions, followed by hydration with an ethanol concentration gradient. Hematoxylin and eosin staining (Servicebio, cat#: G1076) was performed to visualize nuclei and cytoplasmic structures in the sections. Morphological changes of the lung tissue were observed and photographed using a microscope. To quantify the severity of lung injury, scoring was blindly evaluated according to the Smith scoring system (21). Edema, alveolar inflammation, interstitial inflammation, alveolar hemorrhage, interstitial hemorrhage, lung atelectasis, and necrosis were scored on a scale ranging from 0 to 4 based on their extent: no injury received a score of 0; involvement of 25% visual field received a score of 1; involvement of 50% visual field received a score of 2; involvement of 75% visual field received a score of 3; complete involvement across the entire visual field received a score of 4. Two independent randomized visual fields from each mouse were assessed with scores ranging from minimum possible (0) to maximum possible (28).

### 2.6 Immunohistochemistry staining

The lung tissue sections from mice were cut into 4 μm slices after being embedded in paraffin. After antigen retrieval and washing, endogenous peroxidase activity was blocked with a 3% H_2_O_2_ solution in methanol for 20 min at room temperature away from light, followed by washing with 1×PBS. The sections were then incubated with QuickBlock™ Blocking Buffer for Immunol Staining (Beyotime, cat#: P0260) for 15 minutes and stained overnight at 4°C with a primary antibody against PD-L1 (diluted 1:200, Proteintech, cat#:66248-1-Ig). Subsequently, HRP-conjugated goat anti-mouse secondary antibody (diluted 1:5000, Bioworld, cat#: BS20242-Y) was added to incubate the slices for one hour at room temperature. Next, color reaction was developed using diaminobenzidine (Servicebio, cat#: G1212), and nuclei were counterstained with hematoxylin (Servicebio, cat#: G1004).

### 2.7 The lung wet/dry weight ratio

After the lung tissue was completely removed, it was rinsed with normal saline, blotted dry with filter paper, and weighed to determine its wet weight. The tissue was then dried in a 60°C oven for 48 hours and reweighed to obtain its dry weight. Finally, the wet-to-dry weight ratio was calculated.

### 2.8 Western blot

Cells were collected and lysed with RIPA lysis buffer containing 1× proteases/phosphatase inhibitors cocktail (Solarbio, cat#: P6730, cat#: P1260), followed by a 15-minute incubation on ice. The lysates were then centrifuged at 4 °C for 10 minutes at 14,000 rpm. Protein samples (20 μg) were separated on 10% SDS-PAGE gels and transferred to hydrophobic PVDF transfer membranes. Blotted membranes were blocked and incubated overnight with the following primary antibodies: Mouse anti-STAT1 (diluted 1:2000, Abcam, cat#: ab281999), rabbit anti-JAK2 (diluted 1:1000, Abcam, cat#: ab108596), rabbit anti-p-JAK2 (diluted 1:1000, Abcam, cat#: ab32101), rabbit anti-p-STAT1 (diluted 1:2000, Abcam, cat#: ab109461), mouse anti-PD-L1/CD274 (diluted 1:2000, Proteintech, cat#: 66248-1-Ig) and mouse anti-GAPDH (diluted 1:1000, Goodhere biotech., cat#: AB-M-M001). After washing the blotted membranes four times with TBST buffer, they were incubated at room temperature for one hour with suitable secondary-HRP conjugated antibodies as follows: Goat anti-rabbit IgG(H+P) HRP(diluted 1:5000, Bioss, cat#: bs-0295G-HRP) and Goat ant-mouse IgG(H+P)HRP (diluted 1:5000, Bioworld, cat#: BS20242-Y). After washing the blots four times with TBST buffer, the blots were developed using immobilon western HRP substrate(Merck, catalog number WBKLS01001)and visualized by exposures using AmershamTM Imager680(Gene Ray Electric company USA). Finally, the integrated density bands were quantitatively analyzed using Image J software. The signal of the target protein in each lane was normalized by dividing it with the signal value of the internal loading control in that lane.

### 2.9 Immunofluorescence

For the detection of p-JAK2 and p-STAT1 expression, MH-S cells were treated with LPS (1 μg/ml) for 30 min, with or without a one-hour pretreatment with CEP-33779 (3 μM). To detect PD-L1 protein expression, MH-S cells were treated with LPS (1μg/ml) for 24 hours, with or without a two-hour pretreatment with CEP-33779. After treatment, cells were fixed in 4% paraformaldehyde for 15 min and washed three times in 1×PBS. Subsequently, cells were permeabilized for 10 minutes using a 0.1% solution of Triton X-100 (Beyotime, cat#: P0096), except for proteins localized to the cell membrane which did not require any permeabilization treatment. Following this, cells were blocked in QuickBlock™ Blocking Buffer (Beyotime, cat#: P0260) for 30 minutes. Cells were then incubated overnight at the indicated dilution in Immunol Staining Primary Antibody Dilution Buffer (Beyotime, cat#: P0103) containing rabbit anti-p-JAK2 antibody (diluted 1:1000; Abcam, cat#: ab32101), rabbit anti-p-STAT1 antibody (diluted 1:100; Cell Signaling Technology, cat#: 9177), mouse anti-PD-L1/CD274 antibody(diluted 1:100; Proteintech,cat#: 66248-11-Ig), and rabbit anti-CD11c antibody(diluted l:l00; Signalway Antibody,cat#: C91504FITC). Slides were washed in l×PBS and stained using secondary antibodies diluted at a ratio of l:200 in SignalUp™ Secondary Antibody Dilution Buffer (Beyotime, cat#: P0278) with Goat Anti-Rabbit IgG FITC (Servicebio, cat#: GB22303) and at a ratio of l:500 in SignalUp™ Secondary Antibody Dilution Buffer with Goat Anti-Mouse IgG Cy3(Servicebio, cat#: GB21301). Slides were washed again in l×PBS, re-stained with DAPI (Servicebio, cat#: G1012) for nuclear staining, and incubated for 10 min at room temperature away from light. Finally, the Mounting Medium. antifading (Solarbio.cat#: S2100) was applied to the cells before adding the coverslip. All digital images were acquired on a Zeiss laser confocal microscope LSM980 at Guangzhou Medical University.

### 2.10 Enzyme-linked immunosorbent assay

The levels of TNF-α and IL-6 in BALF were measured using Mouse TNF-α ELISA KIT (bsk12002) and Mouse IL-6 ELISA KIT (bsk12004) from Bioss, Beijing, China. The measurements were performed according to the manufacturer’s instructions. The optical density at 450 nm was determined using a microplate reader (Thermo Fisher Scientific, MA, USA), with two replicates for each sample.

### 2.11 Phagocytosis

MH-S cells were plated onto cell slides in a 12-well cell culture plate and divided into three groups: Control group; LPS group (1 μg/ml); LPS + Solvent group; LPS+CEP-33779 group (3000 nM). The groups were treated for 24 hours, followed by discarding the culture medium and adding latex beads-rabbit IgG-FITC complex to the cell culture at a volume ratio of 1:400. After 2 hours, the phagocytic ability of MH-S cells was determined using confocal microscopy and phagocytosis index. The latex beads rabbit IgG FITC Complex was directly added to the preheated complete culture medium at a final dilution of 1:400. After thorough mixing, 600 μl of the mixture was added to each well and incubated in a 37°C and 5% CO_2_ incubator for 2 hours. Subsequently, the culture medium and unbound fluorescent microspheres were gently washed with measuring buffer, fixed with 4% paraformaldehyde for 15 minutes, and then cleaned with measuring buffer to remove residual paraformaldehyde. A brief incubation (1-2 minutes) with trypan blue quenching solution followed by washing with measuring buffer removed FITC fluorescence that only binds to the surface. Residual trypan blue was removed by washing again with measuring buffer. Next, the cells were restained with DAPI for 10 minutes, washed three times, and observed using a Zeiss laser confocal microscope LSM980. The Phagocytosis index (PI) was calculated as the number of fluorescent microbeads ingested divided by the total number of macrophages. 200 cells per slide were counted during this process. Each condition was repeated at least three times in separate experiments.

### 2.12 Statistical analysis

The statistical analysis was conducted using GraphPad Prism software version 9.0.0 (GraphPad Software, San Diego, CA). Comparisons between groups were analyzed using one-way ANOVA with Bonferroni posttest when there are more than 2 groups, as indicated in the figure legends. The independent samples T-Test is utilized to compare the difference in means between two separate sets of data. All experiments were repeated at least three times, and the data were presented as mean ± standard error (Mean ± SE). A P-value less than 0.05 was considered statistically significant.

## 3 Results

### 3.1 The expression of PD-L1 on alveolar macrophages is upregulated in LPS-induced ALI mice

**Figure 1:**
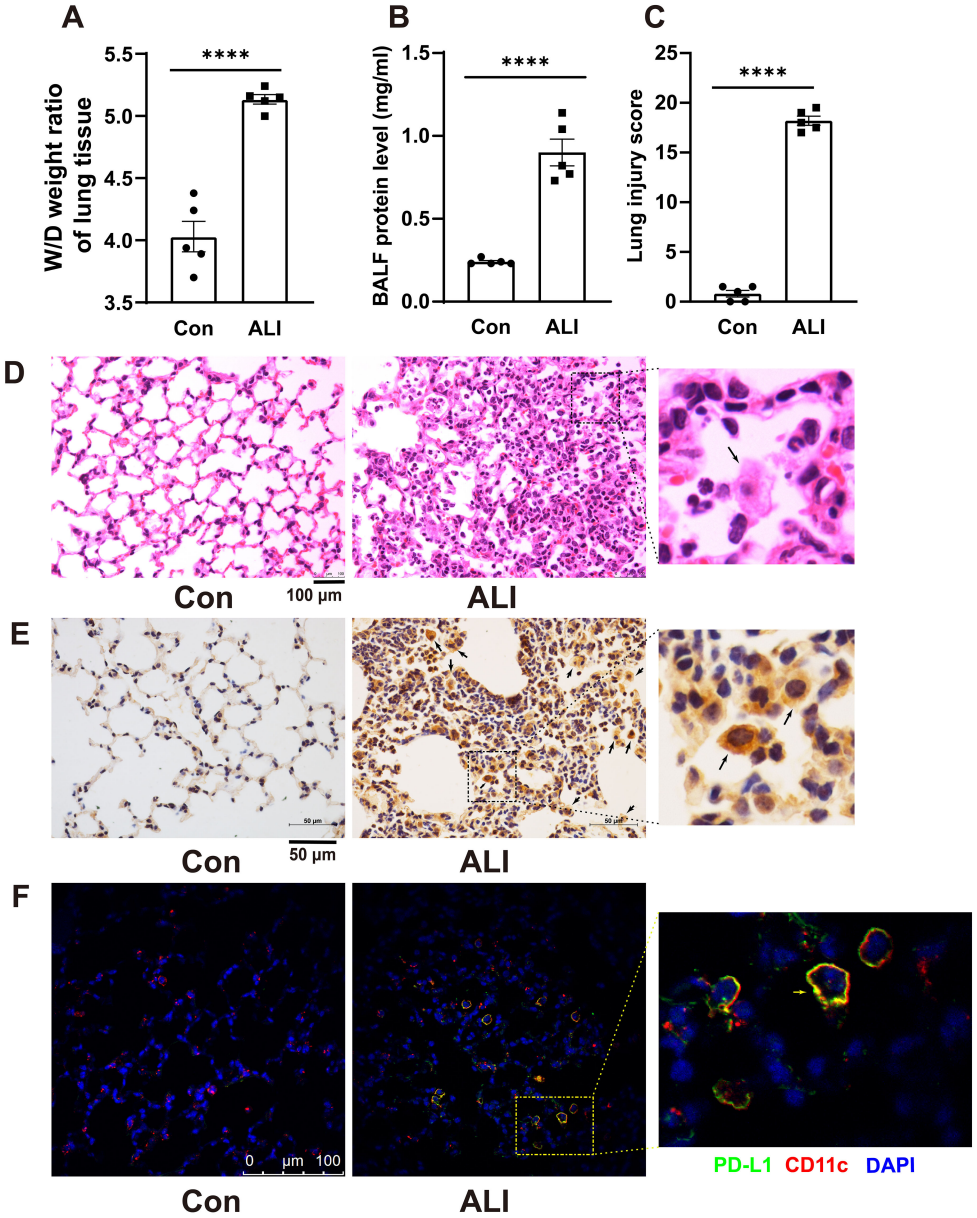
Immunohistochemistry revealed an increased expression of PD-L1 on AMs in the lung tissue of a mouse model with ALI. **(A)** The wet-to-dry weight ratio of lung tissues was measured in both the control group and the ALI model group. **(B)** Protein concentration in BALF was quantified. **(C)** Lung injury score was determined. **(D)** Representative photomicrographs of lung tissues stained with hematoxylin and eosin (H&E) were captured. **(E, F)** The expression level of PD-L1 on AMs in lung tissue was evaluated by IHC and double immunofluorescence staining. The results showed that the expression of PD-L1 on inflammatory cells in the lung tissue of ALI mice was significantly increased, and the expression of PD-L1 on AMs was the strongest. Scale bars indicate 100 μm, and 50 μm. The data are presented as Mean ± SE with n = 5 samples per group. Statistical analysis showed ****P < 0.0001.

The changes in PD-L1 expression in AMs of mice with acute lung injury remain unclear. Therefore, we conducted immunohistochemistry staining to investigate the alterations in PD-L1 expression in lung sections of LPS-induced mice. Acute lung injury was induced by intratracheal instillation of LPS (5 mg/kg) for 48 hours in BALB/c mice. The severity of lung injury was assessed through W/D weight ratio, protein analysis of BALF, HE staining of lung sections, and a lung injury score. As shown in **Figures 1A–C**, these parameters were significantly higher in LPS-induced ALI mice than in the control group. H&E staining revealed characteristic pathological changes associated with LPS-induced acute lung injury. Control mouse lungs in **Figure 1D** exhibited thin alveolar walls with most alveoli devoid of cells. Conversely, ALI mice displayed thickened alveolar septa, collapsed alveoli, and increased inflammatory cell infiltration within interstitial lung tissue and alveoli. Immunohistochemical staining (**Figure 1E**) demonstrated a substantial upregulation of PD-L1 expression on lung slices from ALI mice with exacerbated pulmonary damage, particularly in AMs compared to control mouse lungs. The results of dual immunostaining with PD-L1 and AMs marker CD11c further indicated that PD-L1 was predominantly expressed in AMs (**Figure 1F**).

### 3.2 The JAK2 inhibitor CEP-33779 diminishes the activation of JAK2-STAT1 and PD-L1 upregulation on BALF AMs in LPS-induced ALI mice

**Figure 2:**
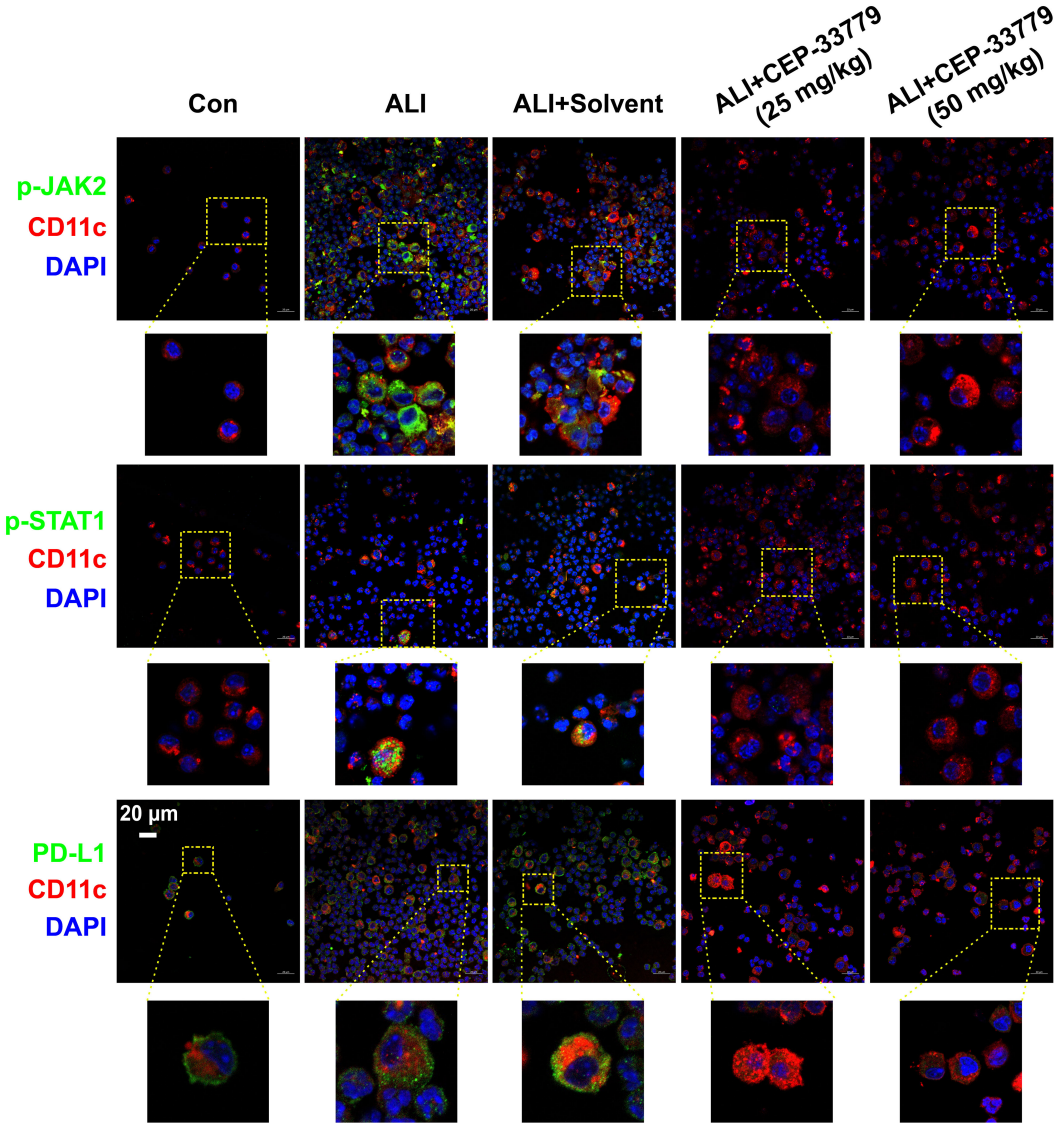
CEP-33779 concentration-dependently suppressed the activation of the JAK2-STAT1 pathway and upregulation of PD-L1 on freshly isolated AMs in an LPS-induced ALI mouse model as shown by immunofluorescence staining. Cells in BALF were separated from each group of mice for immunofluorescence staining, and the expression of p-JAK2, p-STAT1, and PD-L1 was visualized using laser confocal microscopy. Blue fluorescence corresponds to DAPI-stained nuclei; Red fluorescence represents CD11c stained with CD11c antibodies, marking alveolar macrophages. Green fluorescence indicates staining for phosphorylated JAK2 antibodies, phosphorylated STAT1 antibody and PD-L1 antibodies respectively. All laser confocal microscopy photos were captured using a 40x oil mirror. Scale bars indicate 20 μm.

To determine whether LPS induces the phosphorylation of JAK2 (pJAK2) and STAT1 (pSTAT1) and the upregulation of PD-L1 on freshly isolated BALF AMs *in vivo*, mice were challenged with LPS and treated with the JAK2 inhibitor CEP-33779 for 48 hours. Subsequently, fresh cells isolated from BALF were then subjected to immunofluorescence analysis using a confocal microscope to assess JAK2 and STAT1 activation and PD-L1 expression. As illustrated in **Figure 2**, AMs from the BALF of LPS-induced ALI mice exhibited elevated levels of pJAK2, pSTAT1, and PD-L1 expression at 48 hours compared to cells from control mice. In contrast, treatment with the JAK2 inhibitor CEP-33779 abrogated LPS-induced pJAK2-pSTAT1 and PD-L1 overexpression on freshly isolated BALF AMs. These findings demonstrate that concurrent enhancement of JAK2-STAT1 activation and PD-L1 expression occurs in freshly isolated BALF AMs from LPS-instilled ALI mice, while their upregulation is attenuated by *in vivo* treatment with the JAK2 inhibitor CEP-33779.

### 3.3 The activation of JAK2-STAT1 and upregulation of PD-L1 induced by LPS are mitigated by a JAK2 inhibitor and STAT1 inhibitor in MH-S cells

**Figure 3:**
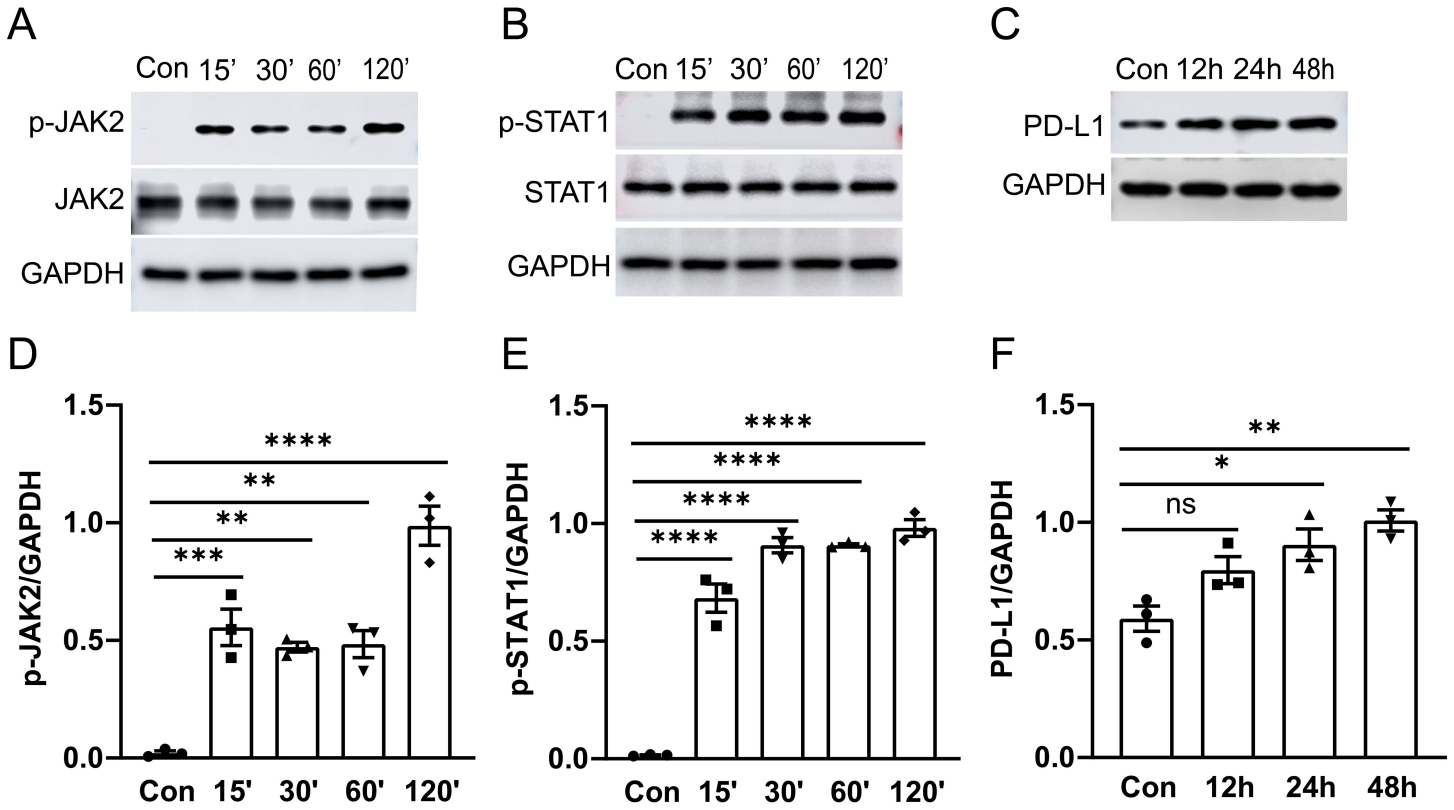
The activation of the JAK2-STAT1 signaling pathway and PD-L1 expression in MH-S cells was induced by LPS. MH-S cells were stimulated with LPS (1 μg/ml) for different durations: 0, 15, 30, 60, and 120 minutes. Western blot analysis was performed to detect the phosphorylation levels and total protein levels of JAK2 and STAT1. A significant increase in phosphorylation of JAK2 and STAT1 was observed at 15 minutes after LPS stimulation **(A, B)**. PD-L1 expression was enhanced after LPS stimulation for 24 and 48 hours **(C)**. Quantitative analysis of the intensity of pJAK2, pSTAT1, and PD-L1 bands **(D–F)**. ns, not significant, *P<0.05, **P<0.01, ***P<0.001, ****P<0.0001.

The JAK2-STAT1 axis has been reported to regulate PD-L1 expression in human melanoma cell lines (22). To further investigate the signaling mechanisms underlying PD-L1 upregulation, we conducted western blot and immunofluorescence analysis with confocal microscopy to examine the activation of JAK2/STAT1 and PD-L1 expression in murine alveolar macrophage cell line MH-S cells *in vitro*. MH-S cells were treated with 1 μg/ml of LPS for specified durations, followed by detection of phosphorylation levels of JAK2 and STAT1 as well as PD-L1 expression by western blot analysis. We observed that treatment with LPS induced phosphorylation of both JAK2 (**Figures 3A, D**) and STAT1 (**Figures 3B, E**) from 15 min to 120 min post-stimulation, while upregulation of PD-L1 (**Figures 3C, F**) was observed after 24 hours of stimulation in cultured MH-S cells.

**Figure 4:**
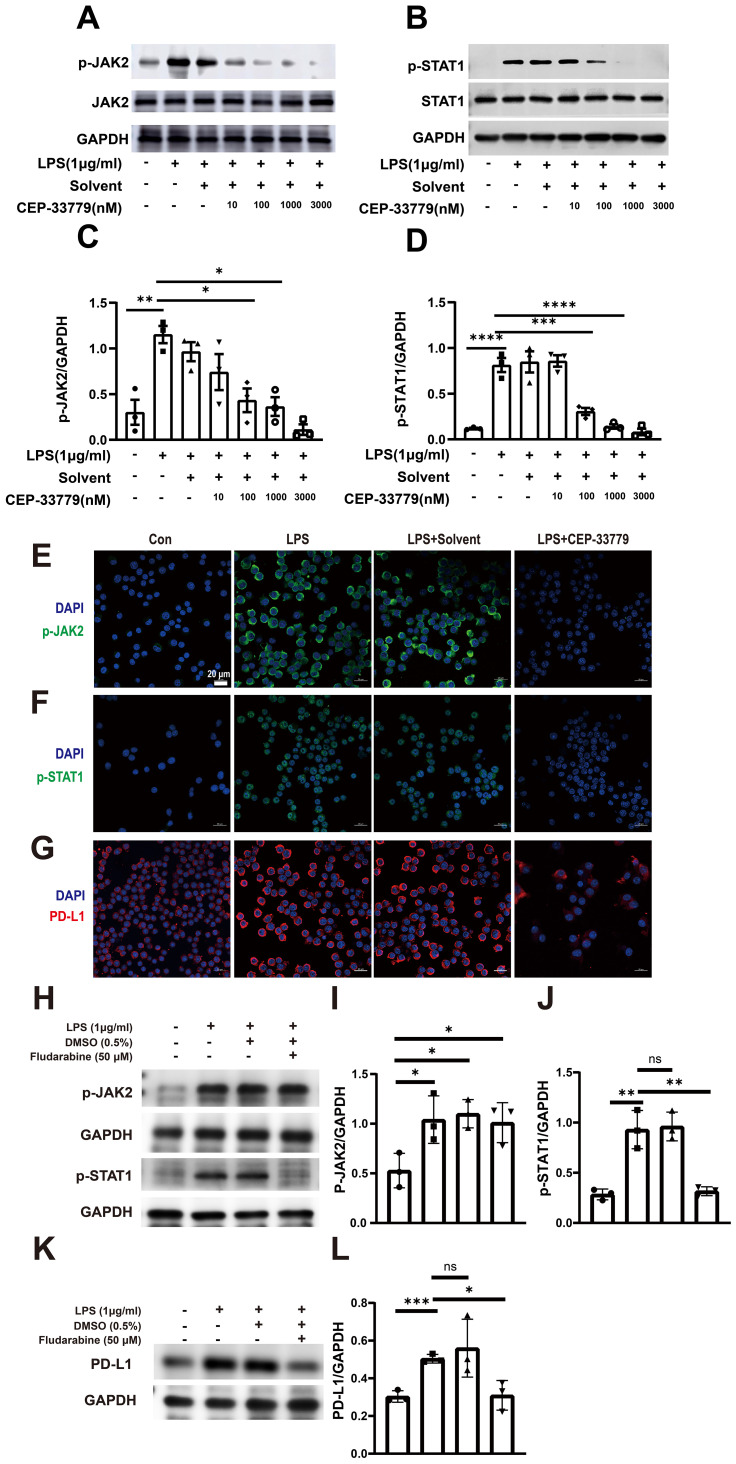
The effect of CEP-33779 and Fludarabine on p-JAK2, p-STAT1, and PD-L1 expression in MH-S cells was analyzed using western blot and immunofluorescence. MH-S cells were pre-treated with varying concentrations (10, 100, 1000, and 3000 nM) of CEP-33779 for one hour before stimulation with 1 μg/ml of LPS for two hours. Subsequently, western blot analysis was performed to detect the phosphorylation levels and total protein levels of JAK2 **(A)** and STAT1 **(B)**. Quantitative analysis was conducted to determine the levels of phosphorylated JAK2, phosphorylated STAT1 and PD-L1 expression **(C, D, I, J, L)**. Representative confocal microscopy images from three independent experiments are shown in panels **(E–G)**. MH-S cells in the LPS group were stimulated with 1 μg/ml LPS for either 2 hours (for p-JAK2 and p-STAT1 expression) or 24 hours (for PD-L1 expression). In the LPS+CEP-33779 group, MH-S cells were pretreated with 3000 nM CEP-33779 for either 1 hour or 2 hours, followed by co-treatment with 1 μg/ml LPS and 3000 nM CEP-33779 for either 2 or 24 hours. Blue fluorescence indicates cell nuclei stained with DAPI; **(E, F)** show green fluorescence representing p-JAK2 and p-STAT1 with nuclear translocation expression respectively; **(G)** shows red fluorescence indicating PD-L1. All laser confocal microscopy photos were taken using a 40x oil mirror. **(H, K)** cells are pretreated with 50 μM of the STAT1 inhibitor fludarabine (MCE, cat number: 21679-14-1) or without it for 24 h. Subsequently, cells are treated with 1 μg/ml LPS for 30 min to detect the levels of p-JAK2 and p-STAT1. In addition, the protein expression levels of PD-L1 are detected by pretreatment with or without 50 μM fludarabine for 24 h followed by treatment with 1 μg/ml LPS for 48 h. The scale bar is 20 μm. The results are presented as Mean ± SE from three independent experiments (ns, not significant, *P<0.05, **P<0.01, ***P<0.001, ****P<0.0001).

Furthermore, inhibition of JAK2 with CEP-33779 diminished both LPS induced activation of JAK2 and STAT1 (**Figures 4A–D**). Subsequently, we employed confocal microscopy to visualize the expression patterns of p-JAK2, p-STAT1, and PD-L1 in MH-S cells. As depicted in **Figures 4E–G**, there was a significant increase in pJAK2 expression following two hours of LPS stimulation; moreover, at this time point, pSTAT1 became highly enriched within the nucleus. Additionally, exposure to LPS for 24 hours resulted in elevated levels of PD-LI expression in MH-S cells. The application of the JAK2 inhibitor CEP-33779 diminished both LPS-induced activation of JAK2-STAT1 pathway and PD-L1 upregulation. Moreover, STAT1 inhibition reduced both LPS-induced activation of STAT1 and PD-L1 upregulation (**Figures 4H–L**). These results demonstrate that LPS stimulation activates JAK2-STAT1 signaling pathway, which mediates PD-L1 upregulation in MH-S cells.

### 3.4 LPS-caused reduction of MH-S cell lines phagocytic activity is significantly improved by CEP33779

**Figure 5:**
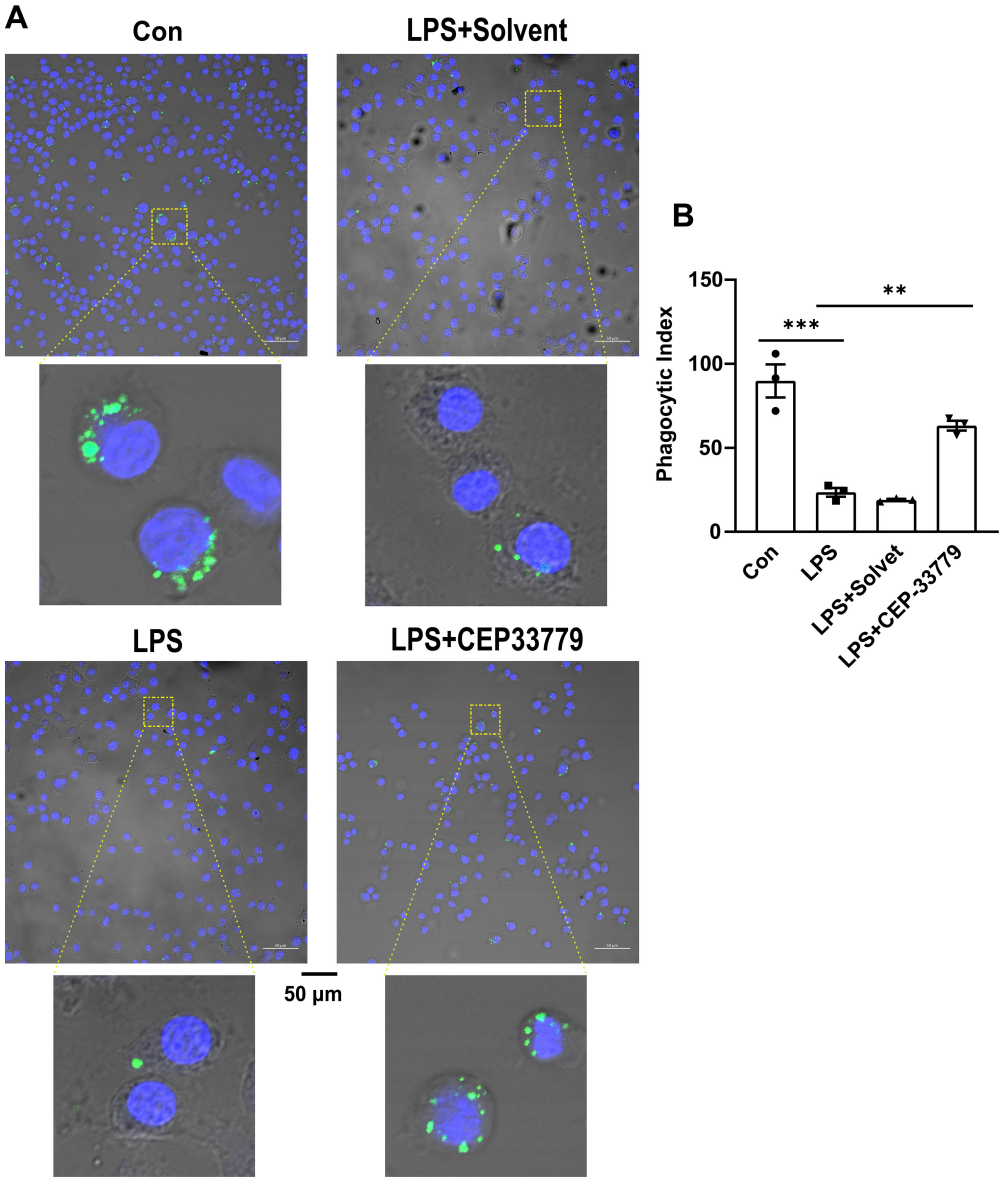
CEP-33779 treatment was able to restore reduction of the phagocytic activity of MH-S cells caused by LPS. **(A)** Representative confocal microscopy images of MH-S cells phagocytosis. MH-S cells were pretreated with 3000 nM CEP-33779 for 2 hours, followed by 1 μg/ml LPS stimulation for 24 hours, then incubated with FITC coupled latex beads for 2 hours. FITC marked latex beads are green. The nucleus of MH-S was stained with DAPI and was blue. The scale bar is 50 μm. All laser confocal microscope photos were taken using a 20x objective lens. **(B)** phagocytic index (PI) of MH-S cells under confocal microscopy. The results are presented as Mean ± SE. N=3, **P<0.01, ***P<0.001.

Our data revealed that LPS-induced activation of JAK2-STAT1 and upregulation of PD-L1 were inhibited by CEP33779 in MH-S cells. Other studies have shown that anti-PD-L1 monoclonal antibody can restore the impaired phagocytic function in monocytes (15). We are curious whether CEP33779 can also restore the impaired phagocytic function of MH-S cells stimulated by LPS. To address this question, we examined the effect of 24-hour treatment with CEP-33779 on the phagocytic function of MH-S cells using latex beads rabbit IgG FITC complex. Compared to a phagocytic index (PI) of 90 in the control group, the PI in the LPS group decreased by 65 (**Figure 5B**). CEP-33779 treatment significantly reversed the inhibitory effect of LPS on MH-S cells phagocytosis, resulting in a strikingly higher number of engulfed green fluorescent latex beads compared to the LPS group (**Figure 5A**). The PI increased by approximately 40% in the LPS+CEP-33779 group compared to the LPS group. In summary, our findings demonstrate that stimulation with LPS leads to a reduced phagocytic index in MH-S cells, while treatment with CEP-33779 improves their phagocytic ability.

### 3.5 The administration of CEP-33779 significantly mitigates LPS-induced lung injury in mice

**Figure 6:**
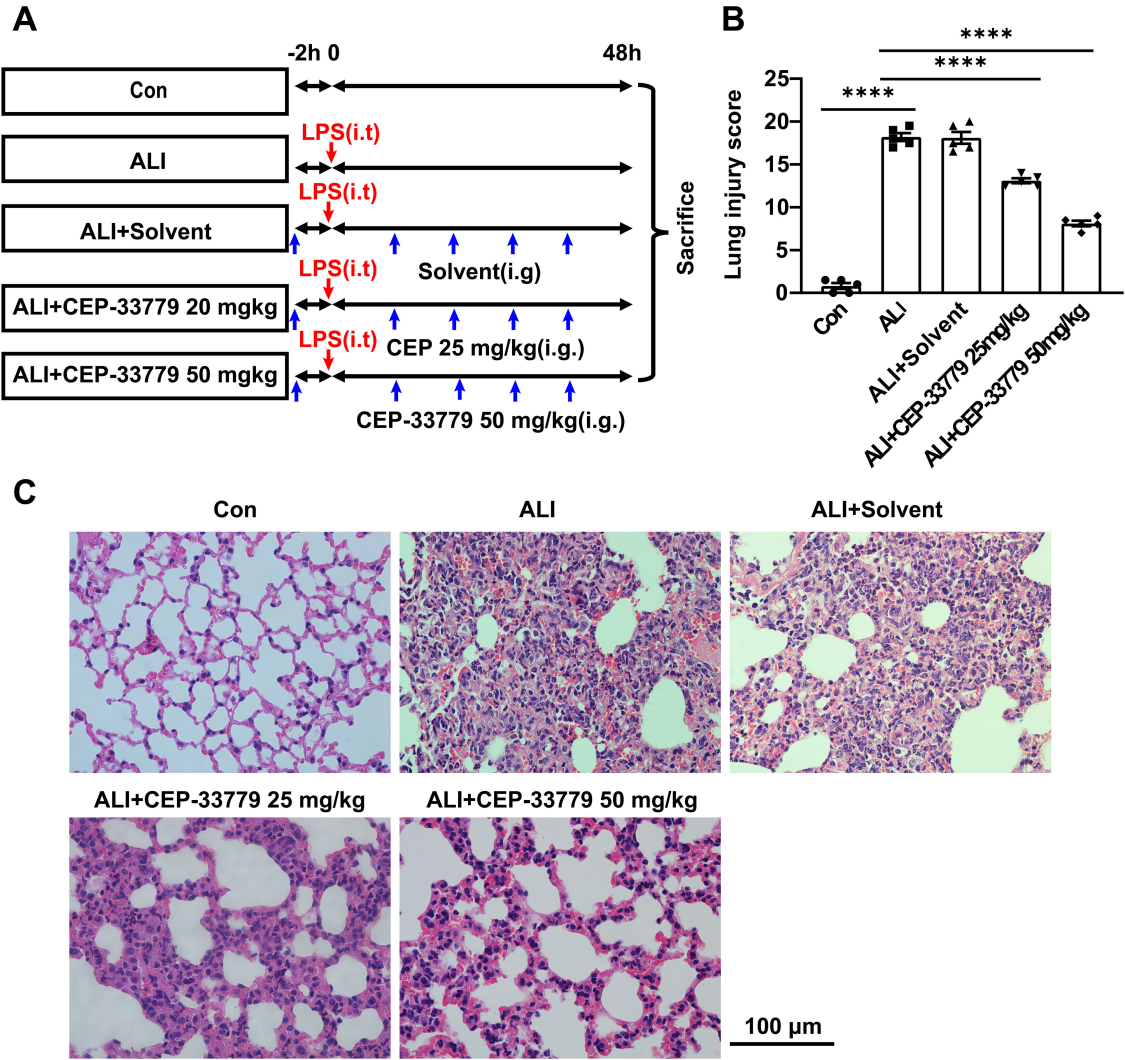
Treatment with CEP-33779 dose-dependently mitigated the extent of lung injury induced by LPS in mice. **(A)** A schematic diagram outlining the experimental plan for animal studies is presented. An ALI model was established by intratracheal instillation of LPS, and CEP-33779 was administered 2 hours prior to LPS instillation and twice daily within 48 hours after LPS instillation. Lung samples were collected from each group after 48 hours of treatment as indicated. **(B)** Assessment of lung injury score based on H&E-stained lung sections. **(C)** Representative photomicrographs showing HE-stained lung tissue slices from Con, ALI, ALI + Solvent, ALI + CEP-33779 25 mg/kg, and ALI + CEP-33779 50 mg/kg groups of mice. The scale bar represents 100 μm with an original magnification at 400 times. Results are presented as Mean ± SE; N=5; ****P<0.0001.

ALI mice were orally administered CEP-33779 at doses of 25 mg/kg and 50 mg/kg, followed by collection of lung tissue for HE staining and assessment of lung injury score to evaluate the pathological changes in the lung tissue (**Figure 6A**). Control group mice exhibited intact lung tissue structure with thin alveolar walls and minimal presence of intra-alveolar macrophages, as depicted in **Figures 6B, C**. In contrast, ALI group mice displayed significant lung tissue damage characterized by diffuse infiltration of inflammatory cells, disruption of normal alveolar structures, notable thickening of alveolar walls, formation of hyaline membrane, and interstitial thickening. Moreover, the ALI group mice showed a substantial elevation in the lung injury score. Conversely, oral administration of CEP-33779 at 25 mg/kg and 50 mg/kg significantly ameliorated these pathological changes in the lung tissue (**Figure 6C**) and dose-dependently reduced the lung injury score (**Figure 6B**). These findings suggest that inhibition of JAK2 kinase improves LPS-induced mouse lung injury.

### 3.6 CEP-33779 reduces inflammatory response in LPS-induced ALI mice

**Figure 7:**
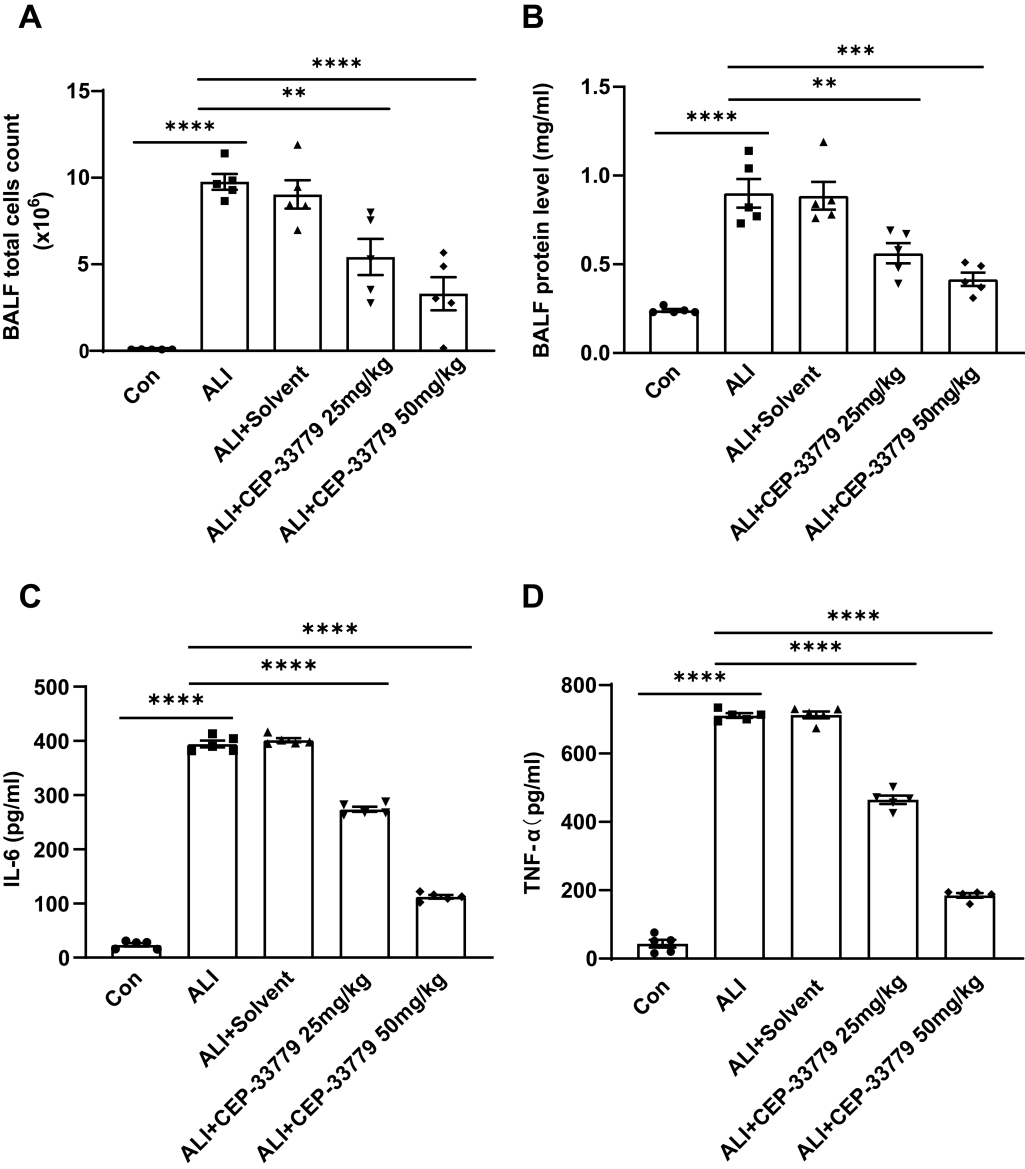
Administration of CEP-33779 attenuates the pulmonary inflammatory response in mice with LPS-induced ALI. **(A)** After 48 hours of treatment, the total cell count in BALF was quantified using a cell counter. **(B)** The concentration of protein in BALF was determined using the BCA method. **(C, D)**. Levels of TNF-α and IL-6 in BALF were measured by ELISA. The results are presented as Mean ± SE. **P<0.01, ***P<0.001, ****P<0.0001. N=5.

To further assess the impact of CEP-33779 on pulmonary inflammatory response, we quantified the total number of cells in mice BALF and measured the concentrations of TNF-α and IL-6 using ELISA. Compared to the control group, both cytokine levels and total cell counts were significantly higher in the ALI group and ALI+PEG300 group. However, treatment with CEP-33779 at doses of 25mg/kg and 50mg/kg significantly reduced TNF-α and IL-6 levels as well as total cell count compared to the ALI group (**Figures 7A, C, D**). Additionally, BALF protein levels were markedly increased 48 hours after LPS instillation in both the ALI group and ALI+PEG300 group using BCA method; however, administration of CEP-33779 led to a significant decrease in total protein concentration within BALF (**Figure 7B**). These findings indicate that treatment with CEP-33779 effectively attenuates LPS-induced pulmonary inflammatory response in mice with acute lung injury.

## 4 Discussion

Our current study demonstrated that the JAK2/STAT1/PD-L1 pathway was up-regulated in freshly isolated BALF AMs from LPS-induced ALI mice. Moreover, treatment with the JAK2-specific inhibitor CEP-33779 restored phagocytic activity of MH-S cell lines and mitigated lung injury in LPS-induced mice by down-regulating the JAK2/STAT1/PD-L1 pathway. These findings suggest that targeting the JAK2/STAT1/PD-L1 pathway may be a promising strategy to restore suppressed AMs phagocytosis and improve acute lung injury.

The development of ALI/ARDS is often triggered by excessive and uncontrolled systemic inflammatory responses to direct or indirect lung injury. Macrophages play a crucial role in the pathogenesis of ALI/ARDS, serving as the first line of defense against pathogenic microorganisms and lung injury (8, 23). PD-L1 has been implicated in immune regulation and appears to be involved in attenuating the overwhelming inflammatory response observed during sepsis (12). We are interested in investigating the alterations of PD-L1 expression on AMs in mice with LPS-induced ALI. Therefore, we initially examined PD-L1 expression on AMs using immunohistochemistry staining and immunofluorescence with confocal microscopy in LPS-induced ALI mice. Our results demonstrated an increased PD-L1 expression on AMs in lung tissue sections, as well as in freshly isolated BALF samples, which correlated with ALI severity. Accumulating evidence suggests that activation of the JAK2-STAT1 pathway occurs in animal models of LPS-induced ALI (16, 19), and this pathway regulates PD-L1 expression in human melanoma cell lines (22). However, it remains unclear whether there is concurrent activation of JAK2-STAT1 signaling and overexpression of PD-L1 on AMs during LPS-induced ALI. This led us to consider whether JAK2 and STAT1 are involved in the upregulation of PD-L1 expression on AMs from ALI mice. Our IF results revealed enhanced phosphorylation levels of JAK2, STAT1, along with increased PD-L1 expression on freshly isolated BALF AMs from LPS-induced ALI mice as well as MH-S cell lines stimulated by LPS. Furthermore, treatment with CEP-33779 reduced phosphorylation levels of JAK2 and STAT1 along with decreased PD-L1 expression on freshly isolated BALF AMs from LPS-induced ALI mice and MH-S cell lines. Besides, treatment with STAT1 inhibitors also resulted in a decrease in LPS-induced levels of STAT1 phosphorylation and down-regulated PD-L1 expression in MH-S cell lines. Overall, these findings demonstrate that JAK2 and STAT1 are activated and potentially mediate the upregulation of PD-L1 on AMs during ALI.

Subsequently, we investigated the potential role of JAK2/STAT1/PD-L1 in AMs’ phagocytic function using a complex of latex beads-rabbit IgG-FITC and fluorescence microscopy. The phagocytic activity of MH-S cell lines was reduced after LPS treatment, but it was restored by CEP-33779. Given that treatment with CEP-33779 downregulated the activation of JAK2 and STAT1 and also suppressed the upregulation of PD-L1 on AMs in response to LPS, and ameliorated the LPS-induced reduction in AMs’ phagocytosis, it is plausible to suggest that the activation of the JAK2/STAT1/PD-L1 signaling pathway may contribute to the decreased phagocytic capacity of AMs induced by LPS. However, the exact role of PD-L1 in AM phagocytosis requires further investigation. Furthermore, histopathological analysis of lung sections stained with HE showed that CEP-33779 significantly improved lung injury in mice with LPS-induced ALI. Treatment with CEP-33779 also resulted in a significant decrease in total protein concentration, as well as levels of TNF-α and IL-6, along with a reduction in total cell count of BALF in mice with LPS-induced ALI. Together, these findings demonstrate that upregulation of JAK2/STAT1/PD-L1 on AMs is associated with reduced phagocytic activity. The downregulation of JAK2/STAT1/PD-L1 on AMs by CEP-33779 improves impaired phagocytic activity and attenuates the pulmonary inflammatory response induced by LPS in ALI mice.

In conclusion, the coincident activation of JAK2/STAT1 and PD-L1 overexpression on AMs was found to be correlated with severe lung injury in LPS-induced ALI mice. Additionally, this also resulted in diminished phagocytic activity in MH-S cell lines. Importantly, blocking the JAK2/STAT1/PD-L1 pathway using the JAK2 inhibitor improved the reduced phagocytic activity of AMs and alleviated lung injury. These findings suggest that targeting the JAK2/STAT1/PD-L1 pathway and enhancing phagocytosis in AMs may offer a novel approach for restoring innate immunity and improving lung injury in ALI.

## Data Availability

The original contributions presented in the study are included in the article/**Supplementary Material**. Further inquiries can be directed to the corresponding authors.
