# Supplementary material for: Repression of JAK2-STAT1 and PD-L1 by CEP-33779 ameliorates the LPS-induced decline in phagocytic activity of alveolar macrophages and mitigates lung injury in mice
Source: Front Immunol. 2024 Nov 26;15:1472425. doi: 10.3389/fimmu.2024.1472425 (PMC11628351; doi:10.3389/fimmu.2024.1472425)

Supplementary Material

Supplementary Material Catalogue

1 **Entire original membrane of Figure 3A 3**

1.1 pJAK2: Replication 1 3

1.2 pJAK2: Replication 2 3

1.3 pJAK2: Replication3 3

1.4 Total JAK2: Replication 1 3

1.5 Total JAK2: Replication 2 4

1.6 Total JAK2: Replication 3 4

1.7 GAPDH: Replication 1 4

1.8 GAPDH: Replication 2 5

1.9 GAPDH: Replication 3 5

2 **Entire original membrane of Figure 3B 6**

2.1 pSTAT1: Replication 1 6

2.2 pSTAT1: Replication 2 6

2.3 pSTAT1: Replication 3 6

2.4 Total STAT1: Replication 1 6

2.5 Total STAT1: Replication 2 7

2.6 Total STAT1: Replication 3 7

2.7 GAPDH: Replication 1 7

2.8 GAPDH: Replication 2 8

2.9 GAPDH: Replication 3 8

3 **Entire original membrane of Figure 3C 9**

3.1 PD-L1: Replication 1 9

3.2 PD-L1: Replication 2 9

3.3 PD-L1: Replication 3 9

3.4 GAPDH: Replication 1 10

3.5 GAPDH: Replication 2 10

3.6 GAPDH: Replication 3 11

4 Entire original membrane of Figure 4A 12

4.1 pJAK2: Replication 1 ... 12
4.2 pJAK2: Replication 2 ... 12
4.3 pJAK2: Replication 3 ... 12
4.4 Total JAK2: Replication 1 ... 13
4.5 Total JAK2: Replication 2 ... 13
4.6 Total JAK2: Replication 3 ... 13
4.7 GAPDH: Replication 1 ... 14
4.8 GAPDH: Replication 2 ... 14
4.9 GAPDH: Replication 3 ... 14
5 Entire original membrane of Figure 4B ... 16
5.1 p-STAT1: Replication 1 ... 16
5.2 p-STAT1: Replication 2 ... 16
5.3 p-STAT1: Replication 3 ... 16
5.4 Total-STAT1: Replication 1 ... 16
5.5 Total-STAT1: Replication 2 ... 17
5.6 Total-STAT1: Replication 3 ... 17
5.7 GAPDH: Replication 1 ... 17
5.8 GAPDH: Replication 2 ... 18
5.9 GAPDH: Replication 3 ... 18

1 Entire original membrane of Figure 3A

1.1 pJAK2: Replication 1

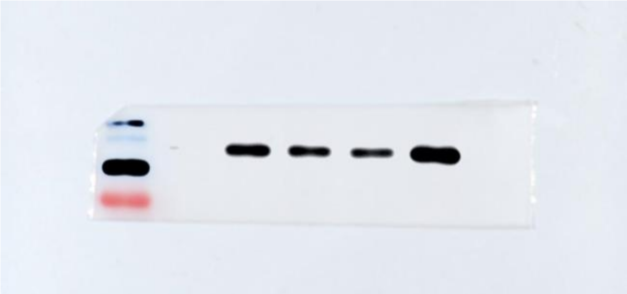

1.2 pJAK2: Replication 2

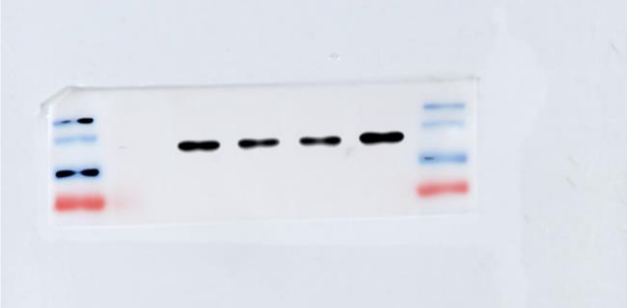

1.3 pJAK2: Replication 3

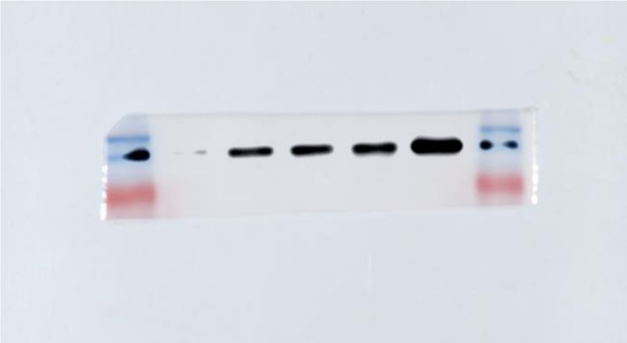

1.4 Total JAK2: Replication 1

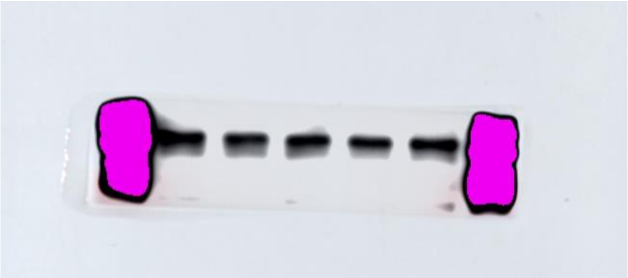

1.5 Total JAK2: Replication 2

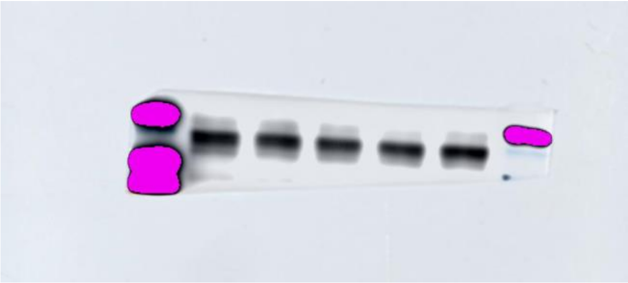

1.6 Total JAK2: Replication 3

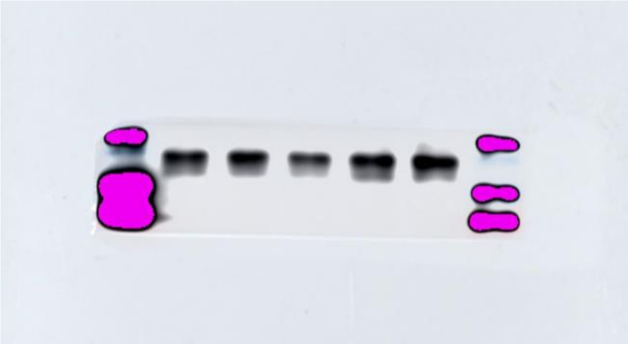

1.7 GAPDH: Replication 1

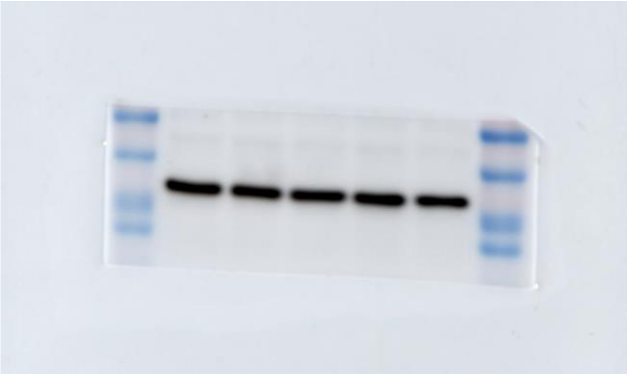

1.8 GAPDH: Replication 2

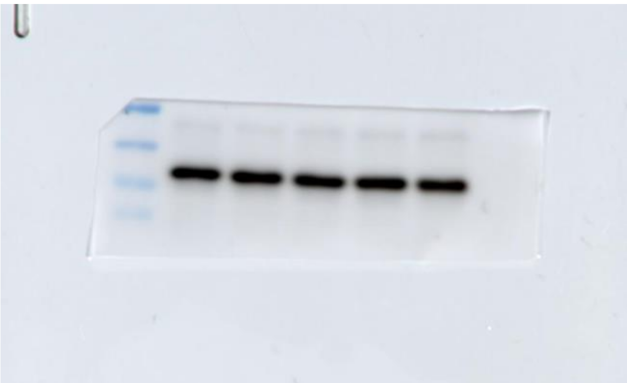

1.9 GAPDH: Replication 3

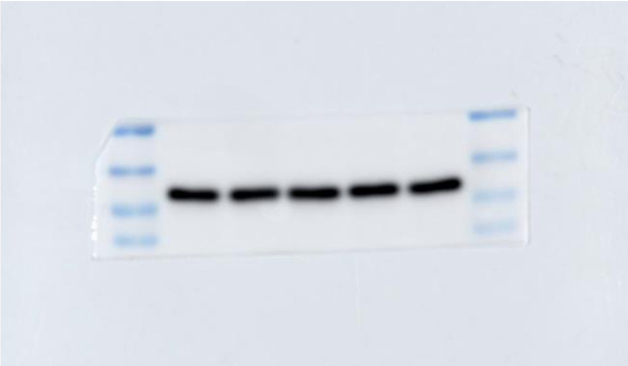

2 Entire original membrane of Figure 3B

2.1 pSTAT1: Replication 1

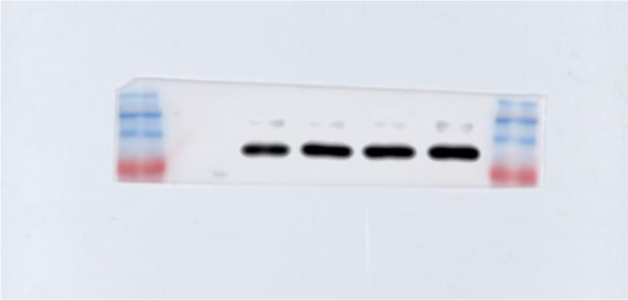

2.2 pSTAT1: Replication 2

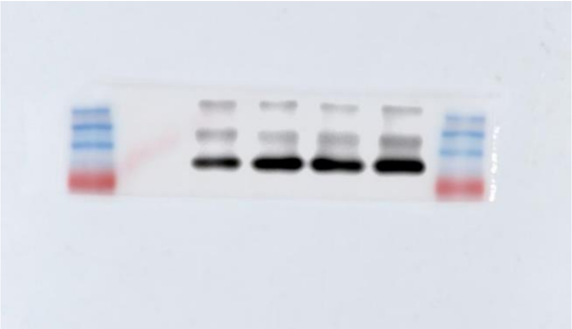

2.3 pSTAT1: Replication 3

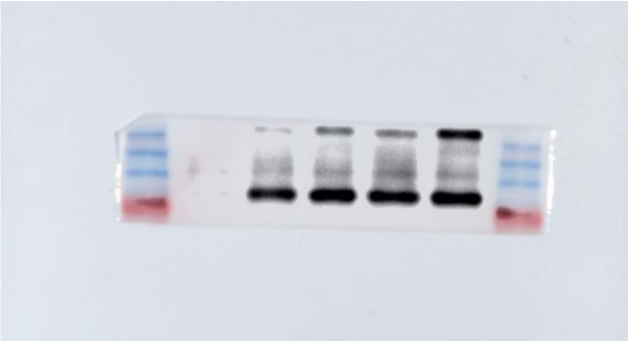

2.4 Total STAT1: Replication 1

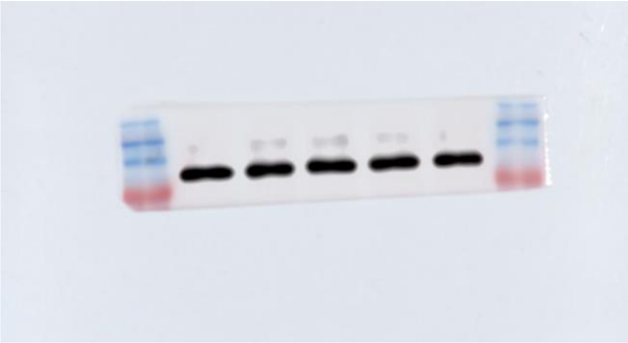

2.5 Total STAT1: Replication 2

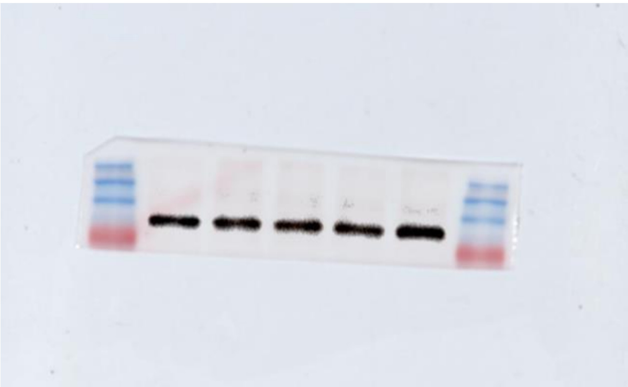

2.6 Total STAT1: Replication 3

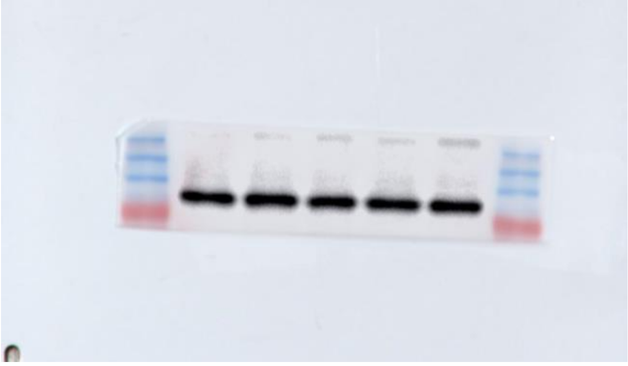

2.7 GAPDH: Replication 1

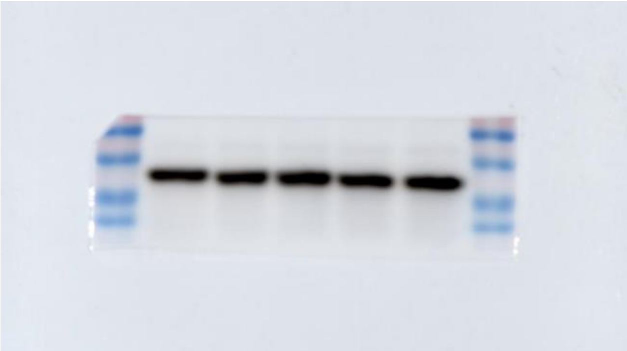

2.8 GAPDH: Replication 2

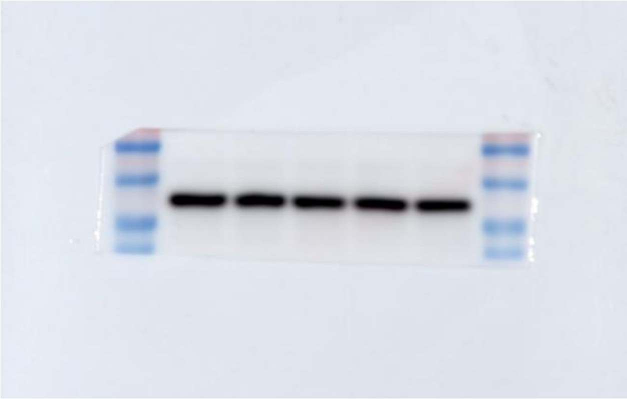

2.9 GAPDH: Replication 3

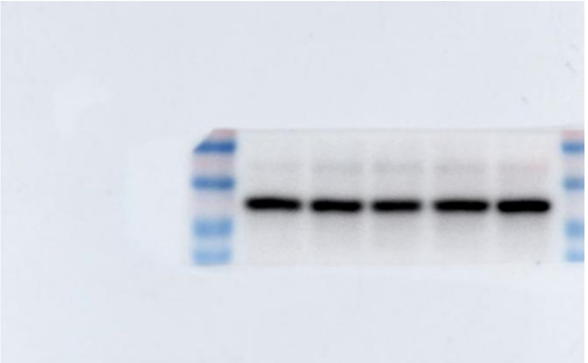

3 Entire original membrane of Figure 3C

3.1 PD-L1: Replication 1

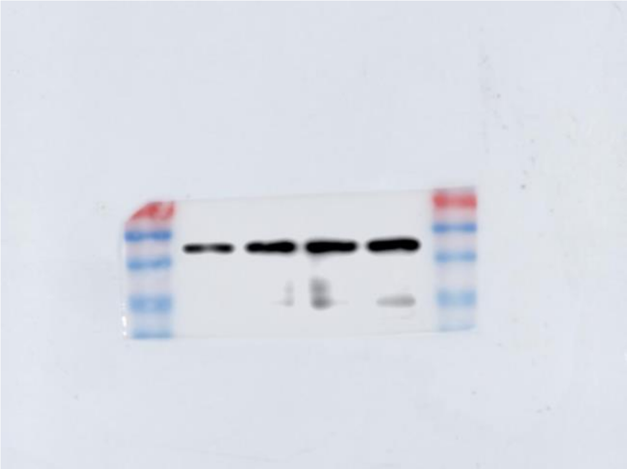

3.2 PD-L1: Replication 2

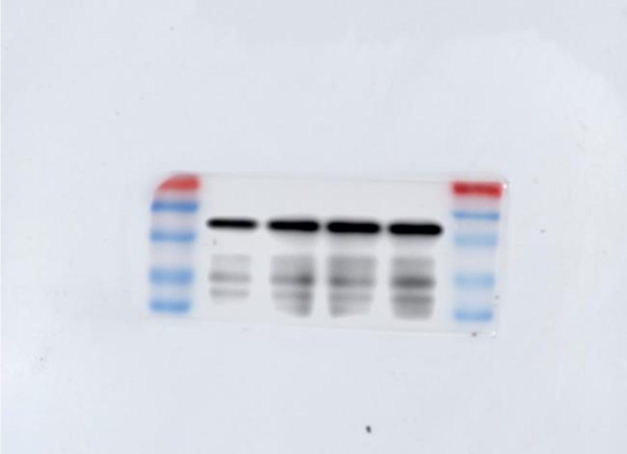

3.3 PD-L1: Replication 3

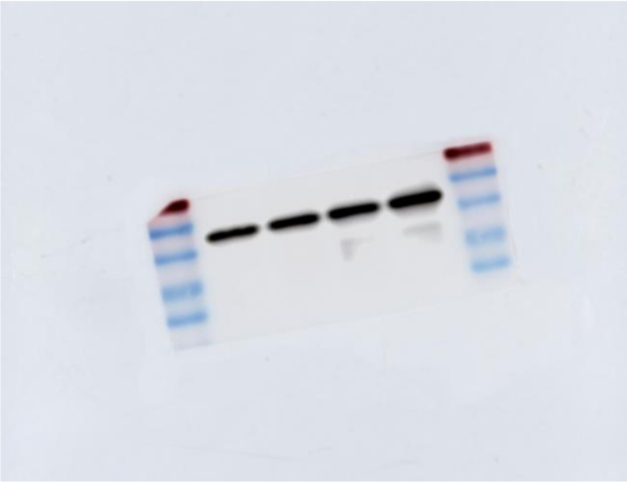

3.4 GAPDH: Replication 1

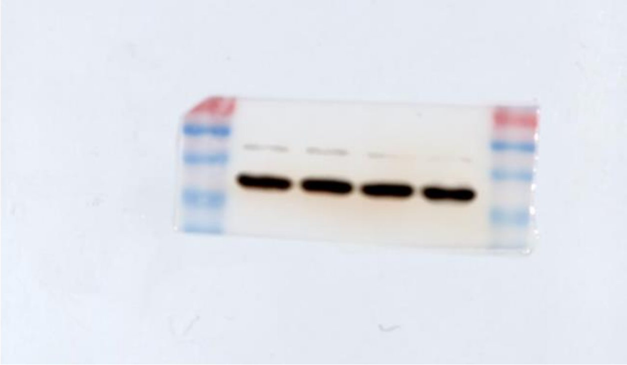

3.5 GAPDH: Replication 2

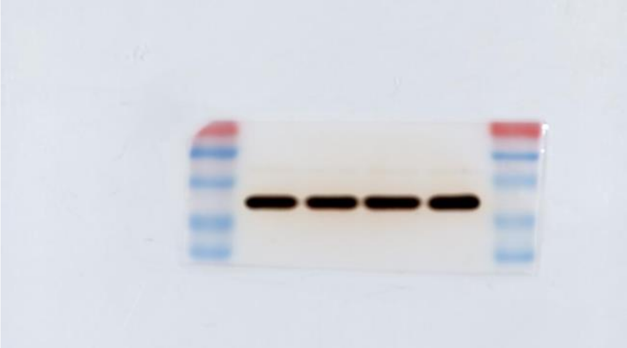

3.6 GAPDH: Replication 3

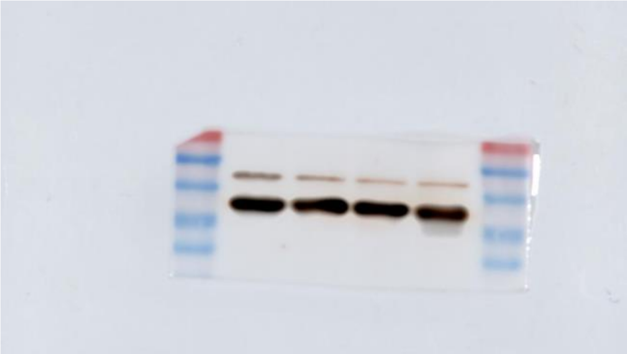

4 Entire original membrane of Figure 4A

4.1 pJAK2: Replication 1

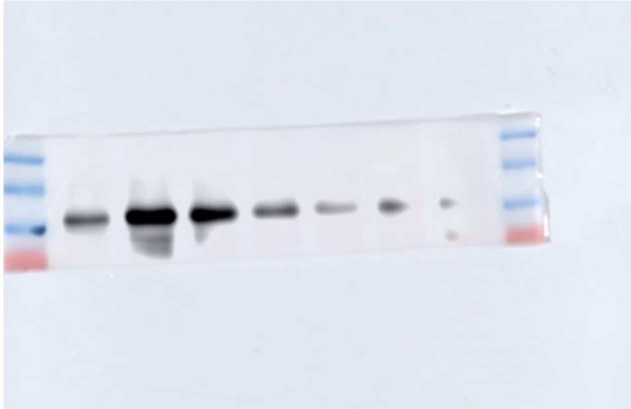

4.2 pJAK2: Replication 2

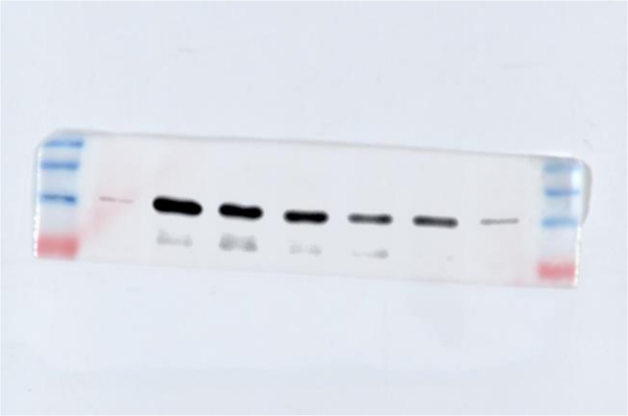

4.3 pJAK2: Replication 3

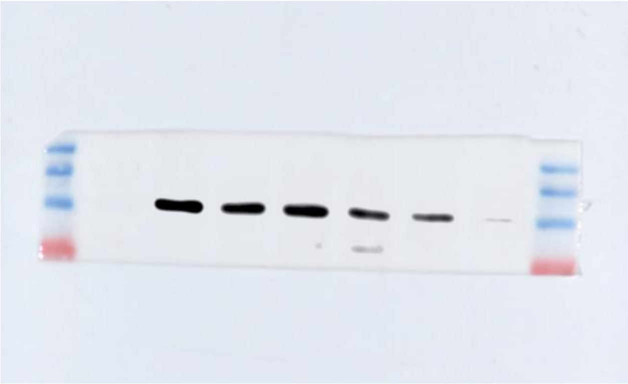

4.4 Total JAK2: Replication 1

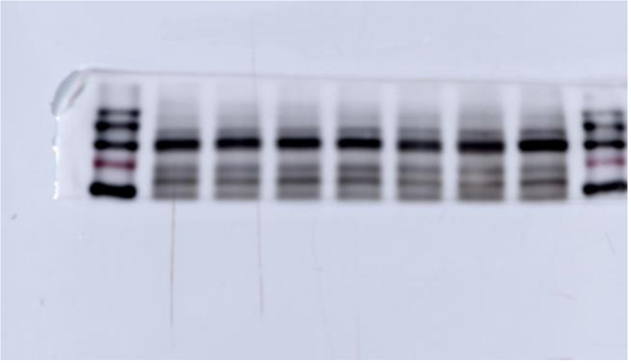

4.5 Total JAK2: Replication 2

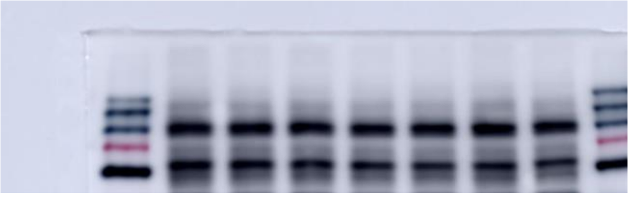

4.6 Total JAK2: Replication 3

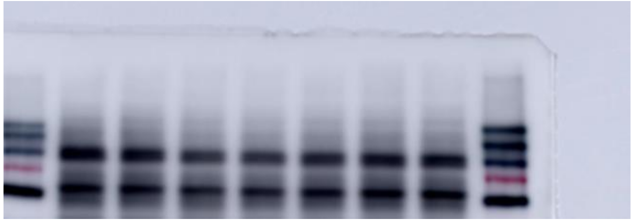

4.7 GAPDH: Replication 1

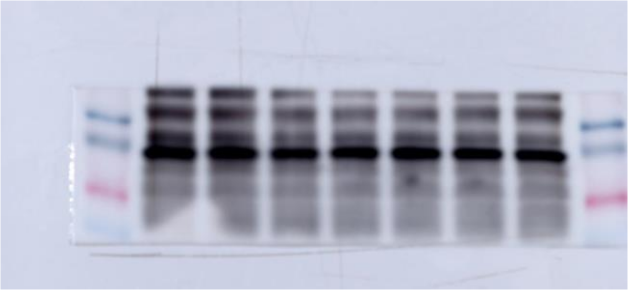

4.8 GAPDH: Replication 2

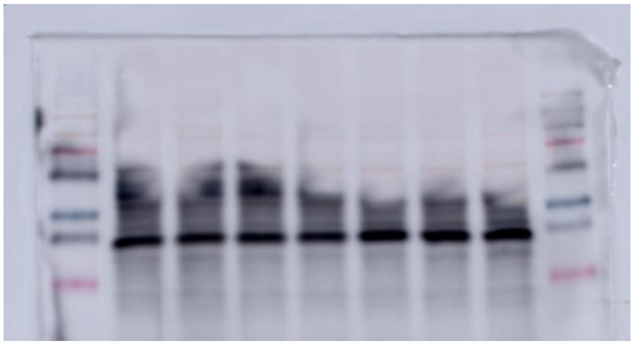

4.9 GAPDH: Replication 3

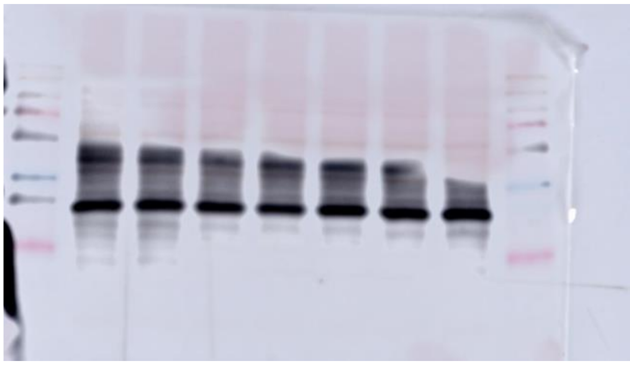

5 Entire original membrane of Figure 4B

5.1 p-STAT1: Replication 1

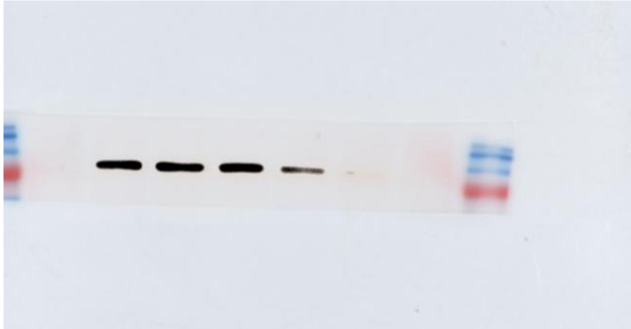

5.2 p-STAT1: Replication 2

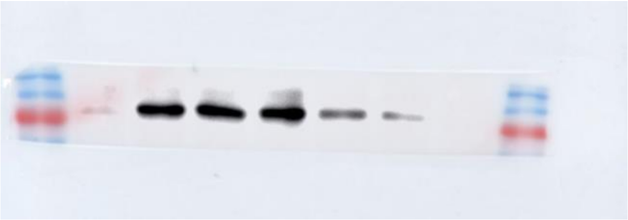

5.3 p-STAT1: Replication 3

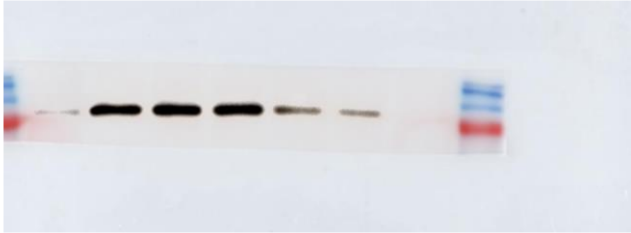

5.4 Total-STAT1: Replication 1

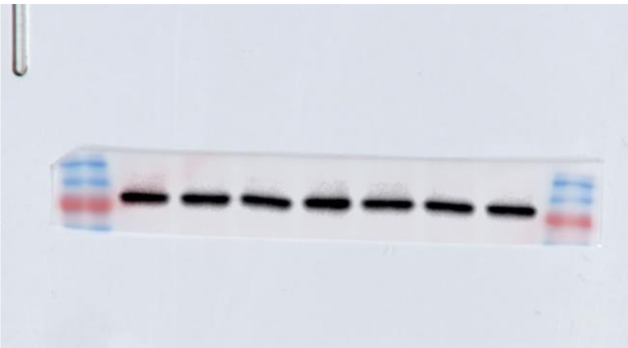

5.5 Total-STAT1: Replication 2

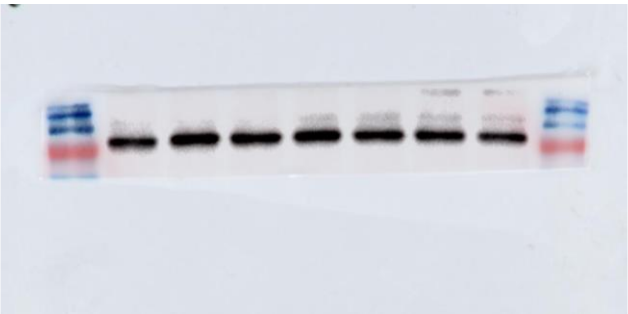

5.6 Total-STAT1: Replication 3

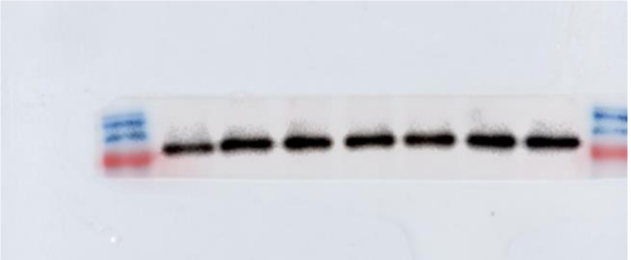

5.7 GAPDH: Replication 1

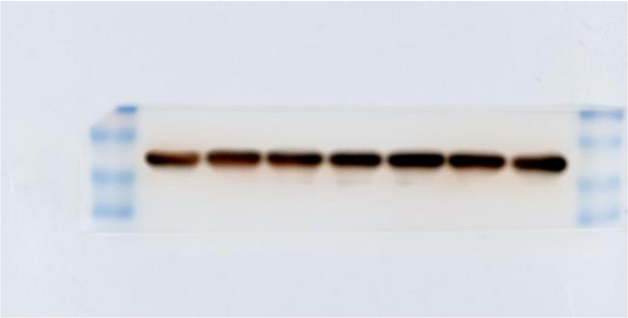

5.8 GAPDH: Replication 2

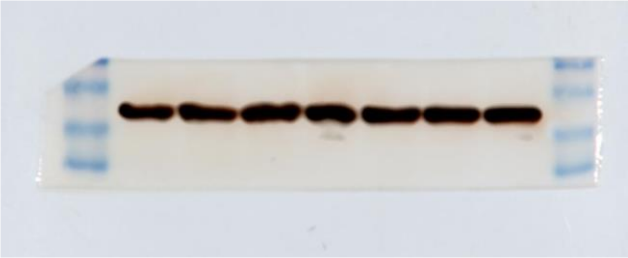

5.9 GAPDH: Replication 3

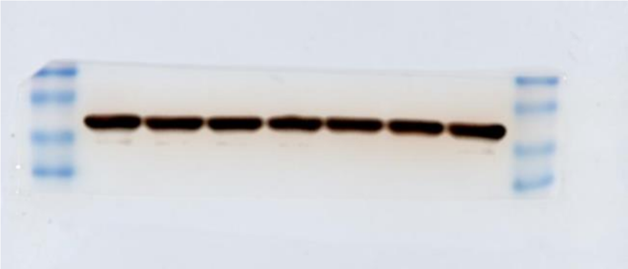

Supplement: Supplementary file 1 [file DataSheet1.pdf]
